# Anti-Inflammatory and Antioxidant Activities of Eugenol: An Update

**DOI:** 10.3390/ph17111505

**Published:** 2024-11-08

**Authors:** Renan Oliveira Silva Damasceno, João Lucas Silva Pinheiro, Lucas Henrique Marques Rodrigues, Rebeca Carneiro Gomes, Allana Brunna Sucupira Duarte, Jeremias Justo Emídio, Lúcio Ricardo Leite Diniz, Damião Pergentino de Sousa

**Affiliations:** 1 Department of Physiology and Pharmacology, Federal University of Pernambuco, Recife 50670-901, Pernambuco, Brazil; renan.oliveirasilva@ufpe.br (R.O.S.D.); joao.pinheiro@ufpe.br (J.L.S.P.); lucas.henriquemarques@ufpe.br (L.H.M.R.); 2 Department of Pharmaceutical Sciences, Federal University of Paraíba, João Pessoa 58051-970, Paraíba, Brazil; rebecagomes123456@gmail.com (R.C.G.); allanabrunna@gmail.com (A.B.S.D.); jeremiasjusto@gmail.com (J.J.E.); 3 National Institute of the Semiarid, Campina Grande 58434-700, Paraíba, Brazil; luciodiniz@yahoo.com.br

**Keywords:** phenylpropanoid, essential oils, natural products, medicinal plants, anti-inflammatory activity, inflammation, cytokines, immunomodulatory activity, asthma, allergy

## Abstract

Medicinal plants are a rich source of bioactive compounds that possess pharmacological properties for preventing and treating inflammation-related diseases. Essential oils is a chemical class that contains many bioactive compounds, such as eugenol, which is capable of inhibiting or modulating the inflammatory response. This natural product emerges as a compound that promotes various biological activities, including antioxidant activity, which makes it useful in the food industry. Recently, its pharmacological applications have also been highlighted. So, this review aims to update and discuss the most recent findings on the anti-inflammatory and antioxidant activities of eugenol, along with its mechanisms of action and therapeutic potential for treating inflammation and oxidative imbalance conditions.

## 1. Introduction

Inflammation is a complex response that helps defend the body against various insults, including infection, injuries, and tissue damage. The inflammatory response can lead to the generation of reactive oxygen species (ROS), resulting in oxidative stress. ROS are highly reactive molecules that, when produced in excess, can overwhelm the antioxidant defenses, causing cellular damage and homeostatic disruptions. Oxidative stress associated with inflammation has been implicated in the pathogenesis of numerous diseases, including cardiovascular, neurodegenerative, and some types of cancer [1,2]. Thus, controlling it is essential for health.

The molecular mechanisms underlying this process involve a variety of signaling pathways with the release of inflammatory mediators and recruitment of immune cells. Understanding these pathways is crucial and provides insights for identifying new drugs and therapeutic targets [3]. In this sense, the nuclear factor-kappa B (NF-κB) signaling pathway plays a fundamental role in the inflammatory response, being responsible for the transcription of pro-inflammatory genes, including tumor necrosis factor-α (TNF-α), interleukin-1β (IL-1β), interleukin-6 (IL-6), and nitric oxide (NO). In general, nonsteroidal anti-inflammatory drugs (NSAIDs) and glucocorticoids are used clinically to treat inflammatory disorders [4]. However, these drugs are associated with side effects (e.g., gastrointestinal ulcers, bleeding, constipation, and abdominal pain) and limited therapeutic efficacy [5,6]. Therefore, it is essential to explore new bioactive compounds.

**Figure 1:**
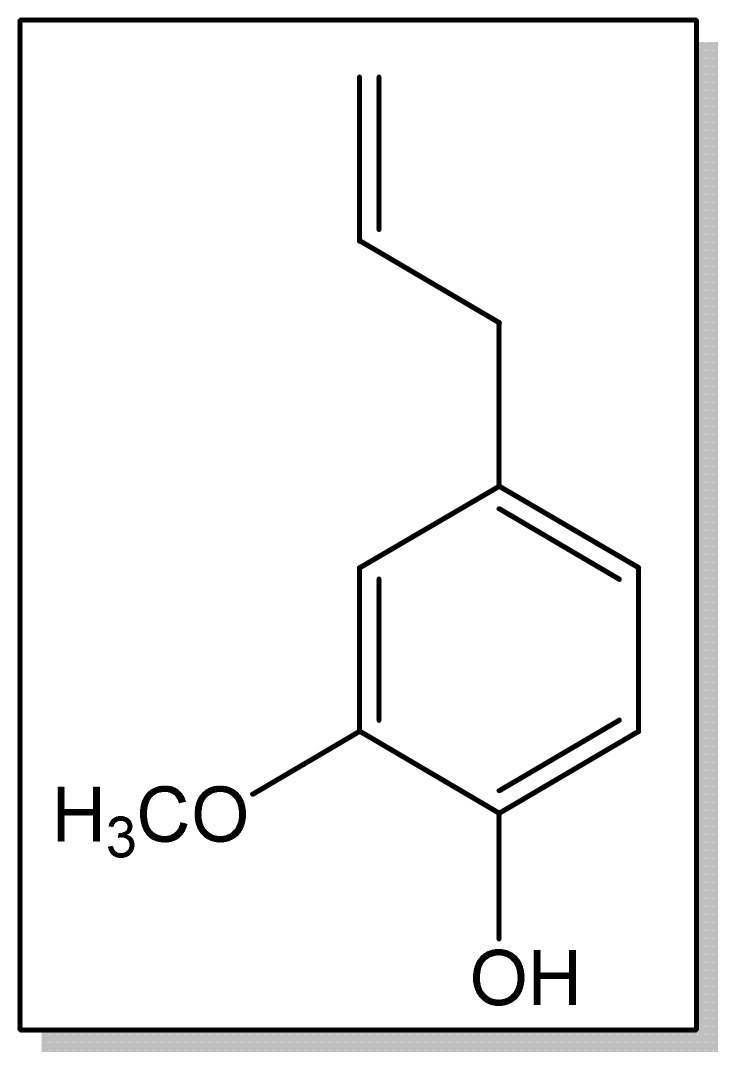
Chemical structure of Eugenol.

Eugenol (4-allyl-2-methoxyphenol, Figure 1) is a phenylpropanoid found in essential oils from a variety of plant families, such as Lamiaceae, Lauraceae, Myrtaceae, and Myristicaceae [7]. It is widely used in foods and cosmetics as a flavoring agent. In recent years, studies have demonstrated that eugenol has anti-inflammatory, analgesic, and antioxidant properties, with potential use in the treatment of cancer, inflammatory bowel diseases, kidney and lung injuries, and osteoarthritis [8,9,10].

This review aims to update and discuss the main recent findings on the use of eugenol and its antioxidant and anti-inflammatory activity in experimental models, and its probable mechanisms of action.

## 2. Overview of the Modulatory Effects of Eugenol on Signaling Pathways of Inflammation and Oxidative Stress

**Figure 2:**
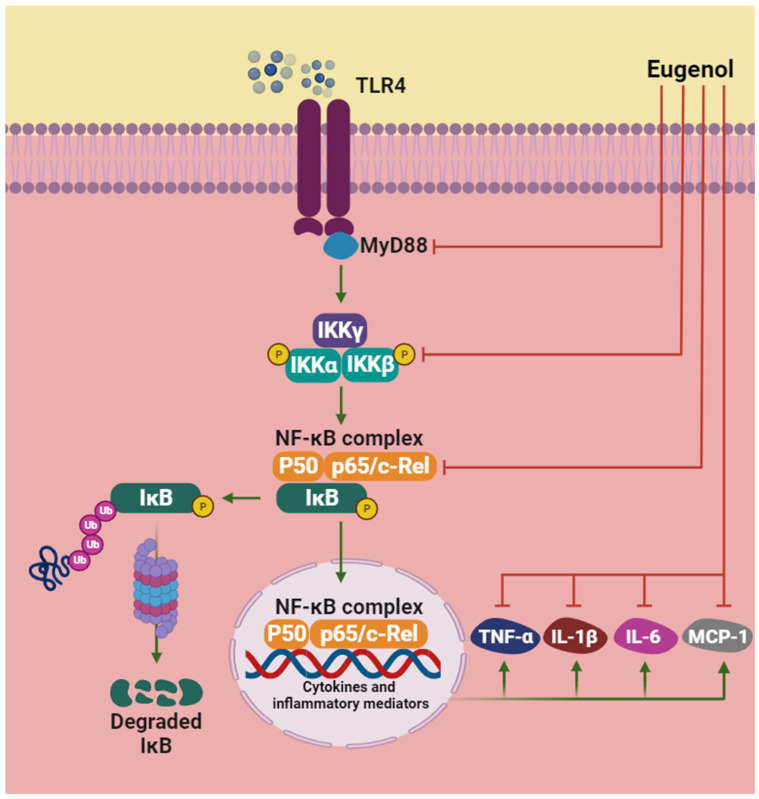
NF-κB signaling is mediated by several receptors and signaling molecules, including TLRs. Eugenol is capable of acting on the inhibition of adapter proteins, such as myeloid differentiation factor 88 (MyD88), suppression of the activation of the IKK complex, and inhibition of the phosphorylation of p65 and IκB, preventing the p50 and p65-RelA heterodimer (family complex NF-κB) to be translocated to the nucleus to induce gene expression. Finally, eugenol also inhibits pro-inflammatory factors related to NF-κB, such as TNF-α, IL-1β, IL-6 and MCP-1. Red lines represent inhibitory action of eugenol. Abbreviations: IL, interleukin; IKK, IκB-kinase; IκB, nuclear factor of kappa light polypeptide gene enhancer in B-cells inhibitor; MCP-1, monocyte chemoattractant protein-1; MyD88, myeloid differentiation factor 88; NF-κB, nuclear factor-kappa B; TNF-α, tumor necrosis factor-alpha.

**Figure 3:**
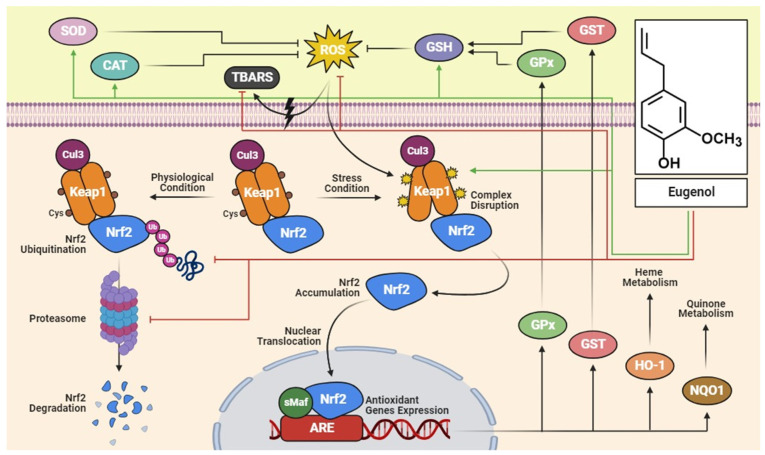
Eugenol can activate antioxidant enzymes such as SOD, CAT, and GSH under conditions of oxidative stress mediated by ROS. It also acts by inhibiting the ubiquitination and degradation of the Keap1-Cul3-Nrf2 complex. Furthermore, eugenol stimulates Nrf2 activation; thus, Nrf2 is released from the Keap1-Cul3 complex and is translocated to the nucleus homodimerized with sMaf proteins and binds to antioxidant response elements (ARE), leading to the transcription of antioxidant genes. Green lines represent the stimulatory action of eugenol, while red lines represent inhibitory action. Abbreviations: ARE, antioxidant response elements; CAT, catalase; Cul3, cullin 3; GSH, glutathione; GST, glutathione S-transferase; GPx, glutathione peroxidase; HO-1, heme oxygenase-1; Keap1, Kelch-like ECH-associated protein 1; Nrf2, nuclear factor erythroid 2-related factor 2; NQO-1, NAD(P)H quinone dehydrogenase 1; ROS, reactive oxygen species; SOD, superoxide dismutase; TBARS, thiobarbituric acid reactive substances.

Eugenol is currently one of the most studied natural polyphenolic compounds due to its wide range of biological activities. Studies using different experimental models have demonstrated the effects of eugenol on cells/tissues and revealed protective effects against arthritic inflammation, pathological lung conditions, and oxidative injury in the liver (Table 1). It also plays a crucial role in regulating systemic inflammation, the response to pathogenic microorganisms, and has neuroprotective and antidiabetic effects (Table 2). The mechanisms associated with the anti-inflammatory and antioxidant effects of eugenol converge on the regulation of transcription factors and pro-inflammatory cytokines, as well as endogenous molecules that constitute the antioxidant defense system (Figure 2 and Figure 3).

### 2.1. Eugenol Reduces Inflammatory Response and Oxidative Stress in Arthritis Conditions

Rheumatoid arthritis (RA) is a prevalent inflammatory joint disease that significantly impacts the well-being of an individual. It is characterized by persistent inflammation, resulting in severe pain, progressive disability, systemic complications, and premature mortality [40]. Studies have demonstrated that immune cells massively infiltrate synovial membranes and represent an important factor in the worsening of inflammatory arthritis by releasing pro-inflammatory cytokines, myeloperoxidase, and ROS [41,42].

Wang et al. (2022) [11] studied the effects of eugenol on fibroblast-like synoviocytes (FLS) from individuals with RA. Cells were stimulated with TNF-α to express an arthritic phenotype. The treatment with eugenol promoted a significant reduction in the growth of these cells, an increased rate of apoptosis, and a decrease in migration, aggressiveness, and inflammatory response. This study also demonstrated that eugenol promoted a reduction in the levels of Vascular Endothelial Growth Factor (VEGF), interleukin-8 (IL-8), and IL-6, indicating a reduction in inflammation and possible modulation of gene transcription. In the Western blot assay, the authors observed increased expression of proteins from the NF-κB signaling pathway (p-IKK, p-IκBα, and p65) when cells were stimulated by TNF-α, while after treatment with eugenol, these levels were reduced after 24 h. Therefore, eugenol has shown promising results for the treatment of RA [11].

Furthermore, Mateen et al. (2019) [12] determined the anti-inflammatory effects of eugenol on peripheral blood mononuclear cells (PBMC) of patients with RA. The results revealed that PBMCs treated with eugenol showed a significant and dose-dependent decline in TNF-α and IL-6 levels monitored for 24 h. In another study, Mateen and colleagues (2019) [13] revealed that eugenol improved the levels of TNF-α, IL-6, and IL-10 in rats with arthritis. This compound also promoted a significant decrease in the levels of ROS, nitric oxide, and biomolecular oxidation markers, and an increase in enzymatic and non-enzymatic antioxidants, suggesting its effectiveness in reducing the severity of arthritis.

Jabbari et al. (2020) [14] investigated chitosan nanoparticles containing eugenol in the healing process of RA in neonatal rats. The data revealed that the nanoparticles downregulated monocyte chemoattractant protein-1 (MCP-1), transforming growth factor beta 1 (TGF-β), and malondialdehyde (MDA). Chitosan nanoparticles containing eugenol also alleviated symptoms of joint inflammation caused by RA. Along this line, studies have suggested that eugenol may be a natural complement and a promising alternative antiarthritic agent. For example, Adefegha et al. (2018) [15] showed that oral treatment with eugenol for 21 days produced an increase in spontaneous movement accompanied by a reduction in inflammatory cell infiltration and tibiofemoral joint edema in rats with carrageenan-induced arthritis. According to the authors, the possible antiarthritic mechanisms may be, at least in part, through the modulation of the activity of the arginase enzyme and the hydrolysis of adenosine nucleotides.

Another possible mechanism involved in the beneficial effects of eugenol in arthritis models may be linked to its antioxidant action. A study developed by Adefegha et al. (2018) [43], revealed that eugenol was able to restore the activity of superoxide dismutase (SOD), catalase (CAT), glutathione peroxidase (GPx), and glutathione S-transferase (GST) in the liver of rats with arthritis. These enzymes are endogenous antioxidant factors and are considered the first line of defense against the formation of free radicals [44]. SOD acts by converting superoxide radicals (O_2_^−^) into molecular oxygen (O_2_) and hydrogen peroxide (H_2_O_2_) which, depending on the concentration, is transformed into H_2_O and O_2_ by CAT or GPx. In parallel, GST catalyzes the conjugation of several substances to make them more water-soluble compounds. Furthermore, eugenol also increased levels of reduced glutathione (GSH), a non-enzymatic antioxidant molecule capable of preventing cellular damage, and decreased the production of thiobarbituric acid reactive substances (TBARS), a product formed from lipid peroxidation, indicating its ability to eliminate free radicals generated in inflammatory arthritis.

### 2.2. Eugenol Appears to Be a Promising Molecule for Controlling Lung Inflammation

Acute lung injury (ALI) is a condition that results in the loss of integrity of the alveolar-capillary membrane, which triggers the recruitment of neutrophils and macrophages, and the release of cytokines, an inflammatory condition that can disrupt the vascular endothelium and epithelium of the lungs [16]. 

Using in vivo and in vitro protocols, a study conducted by Barbosa-de-Oliveira and colleagues (2023) [45] investigated the effects of eugenol on cigarette smoke-induced ALI. Cigarettes contain substances such as heavy metals and aromatic hydrocarbons, which increase ROS levels and activate macrophages, neutrophils, and lymphocytes in the airways, contributing to oxidative imbalance associated with pulmonary edema, increased pulmonary epithelial permeability, and the migration of inflammatory cells to adjacent tissues [46,47]. In this study, in vivo data revealed that the administration of eugenol preserved the lung parenchyma and reduced leukocyte infiltration promoted by cigarette smoke and associated with lower ROS formation, indicating reduced oxidative stress. From in vitro analysis, eugenol reduced the levels of keratinocyte chemoattractant (KC), an immunoregulatory cytokine belonging to the CXC family, in RAM cells exposed to cigarette smoke extract (CSE) [16].

In this line, Bittencourt-Mernak et al. (2021) [17] demonstrated that eugenol reduced pulmonary edema, inflammatory infiltrate, IL-6, and IL-1β levels in bronchoalveolar lavage in mice submitted to acute lung inflammation by lipopolysaccharides (LPS). These data were correlated with a reduction in inflammatory cells positive for inducible nitric oxide synthase (iNOS), matrix metalloproteinase-9 (MMP-9) and tissue inhibitor of metalloproteinases-1 (TIMP-1), collagen content, and 8-isoprostane expression in lung tissue. Eugenol also inhibited the phosphorylation of Jc-Jun-NH_2_ terminal Kinase (JNK), a signaling protein involved in the mitogen-activated protein kinases (MAPKs) pathway.

Using an LPS-induced inflammation model, Hui and colleagues (2020) [18] demonstrated that eugenol suppressed IL-8 levels and the expression of TNF-α in porcine intestinal epithelial cells (IPEC-J2). In addition, eugenol also improved the lung functional and histological changes induced by LPS in animals by inhibiting the release of inflammatory cytokines, including TNF-α, IL-1β, and IL-6 [19].

### 2.3. Eugenol Improves Inflammatory Response and Oxidative Imbalance in Hepatic Disorders

The hepatic system is critical for maintaining physiological homeostasis as it plays a role in detoxification, metabolism, and digestion. However, various pathological conditions can disrupt this equilibrium, leading to inflammatory responses and oxidative imbalances in the hepatic microenvironment.

A study carried by Fathy et al. (2019) [20] demonstrated that treatment with eugenol significantly reduces biochemical, inflammatory, and histopathological changes in rats with carbon tetrachloride (CCl_4_)-induced hepatic fibrosis. This effect is explained by the ability of the eugenol to negatively regulate the expression of pro-inflammatory cytokines (i.e., IL-6, and TNF-α) and the transcription factor NF-kB. Additionally, the combined administration of eugenol and telmisartan (an angiotensin II receptor blocker) had a greater effect than the substances used alone. 

Harb et al. (2019) [21] studied the hepatoprotective activity of eugenol in mice on a diet rich in cholesterol and fat. Eugenol reduced liver weight and lipid accumulation. It also prevented liver injuries and inflammation caused by hypercholesterolemia, reversing hepatic steatosis. These data were corroborated by liver function biomarker results (alanine transaminase [ALT] and alkaline phosphatase), providing a basis for the efficacy of eugenol in ameliorating inflammatory conditions in the liver. Eugenol also negatively regulated the expression of transient receptor potential vanilloid 1 (TRPV1), a channel upregulated in inflammatory states and fatty diseases, while activation of its signaling causes fat accumulation [48]. The authors suggest that chronic consumption of eugenol prevents hypercholesterolemia and fatty liver disease through desensitization of TRPV1 in later stages [21].

The effects of eugenol have also been studied on cadmium-induced liver toxicity in rats [22]. Daily treatment with eugenol significantly reduced TNF-α, IL-6, and NO levels, compared to the cadmium group. It has been postulated that cadmium-induced liver toxicity involves an imbalance of the oxidative status, with intense ROS production and inhibition of antioxidant enzymes [49]. The authors demonstrated that eugenol recovered the SOD, CAT, and GST activity, improved GSH levels, and reduced ROS production and oxidation of hepatic proteins altered by cadmium administration [22]. Thus, these findings support the promising role of eugenol as an anti-inflammatory and antioxidant agent in liver conditions.

### 2.4. Eugenol Possesses a Neuroprotective Effect Against Neuronal Injury

Neurological disorders are one of the main causes of disability and the second leading cause of death worldwide [50]. Many neurodegenerative diseases are caused or aggravated by inflammatory processes, apoptosis, autophagy, or resulting from an oxidative imbalance [51]. These events can also lead to peripheral nerve dysfunction, especially in diabetic neuropathy, causing sensory and motor impairments in patients [52]. As available treatments are often unable to directly treat the cause of the problem, research has focused on finding molecules that suppress the aggravating mechanisms of neurological diseases.

Using an experimental model of streptozotocin-induced diabetes, Alharthy and colleagues (2023) [23] demonstrated that the association between eugenol and isoeugenol, its isomer, increased concentrations of nerve growth factor (NGF), a crucial factor for maintaining the homeostasis of sympathetic and sensory nerves, as it provides axonal growth. As reduced NGF levels in diabetic rats are intrinsically linked to the development of neuropathy, restoration of its levels by eugenol or isoeugenol demonstrates its neuroprotective capacity. The authors also found that both compounds, together or alone, decreased TNF-α concentration in Schwann cells and macrophage, as well as reduced the concentration of TBARS and increased the levels of SOD, CAT, and GSH in the nerve homogenate, presenting maximum antioxidant effect when used in combination. These molecules may contribute to the improvement of nerve function associated with diabetic neuropathy due to their ability to reduce inflammatory and oxidative mediators [23].

Recent studies have suggested that eugenol exerts a neuroprotective effect against neuronal damage via inhibiting the inflammatory response. Zhu et al. (2023) [24] investigated the effects of eugenol in an experimental model of pilocarpine-induced status epilepticus (SE) in mice and showed that eugenol attenuated IL-1β and TNF-α expression, and inhibited NF-κB activation and NLRP3 inflammasome formation in the hippocampus after SE. 

Additionally, studies have also investigated the antioxidant effects of eugenol in models of neurodegenerative diseases. Pontes et al. (2021) [25] administered 6-hydroxydopamine (6-OHDA) into the striatum of adult rats, a neurotoxin widely used to induce models of Parkinson’s disease, as it selectively destroys catecholaminergic neurons by inducing oxidative stress [53]. The authors demonstrated that eugenol restored locomotor performance and reduced immobility time in rats subjected to a 6-OHDA injection. These findings were associated with its antioxidant activity, as eugenol reduced MDA and NO levels in all brain areas analyzed (striatum, hippocampus, and prefrontal cortex) and restored GSH levels. Furthermore, eugenol demonstrated a high capacity to upregulate the expression genes related to antioxidant defenses (SOD, CAT, and GPx) and neurotrophic factors (BDNF, Ntrk2, GFRα1, and GDNF), reinforcing its promising effects against the neurological damage present in Parkinson’s disease [25].

In another study, Adefegha and colleagues (2021) [26] evaluated the neuroprotective effect of eugenol against the oxidative damage characteristic of Alzheimer’s disease in vitro. The results showed that eugenol has a high capacity to scavenge ABTS radicals and ferric reducing power due to the donation of electrons by hydroxyl groups present in its structure. The increase in TBARS levels in brain tissue was significantly suppressed by eugenol, demonstrating its protective effect against the peroxidation lipid of the neuronal membrane. Eugenol also inhibited the activity of cholinesterase (ChE) and monoamine oxidase (MAO), enzymes that degrade neurotransmitters and favor the worsening of neurodegenerative diseases [26]. Together, these results suggest that eugenol is a phytoconstituent that suppresses neuroinflammation and oxidative stress present in neurological diseases and stands out as a promising tool for the treatment of Alzheimer’s disease.

### 2.5. Eugenol Improves Inflammatory and Oxidative Events Associated with Diabetes

Diabetes is a chronic hyperglycemic condition that leads to a group of metabolic changes. Microvascular complications, including retinopathy, diabetic nephropathy, and neuropathy, and macrovascular complications, such as stroke, coronary artery disease, and peripheral arterial disease, are common in diabetes mellitus and require special attention [54]. Inflammation and excessive generation of ROS are etiological factors in the development of insulin resistance, which leads to diabetes [55]. The search for new antidiabetic agents involves molecules capable of regulating the inflammatory and oxidative processes.

Kokabiyan et al. (2023) [27] showed that eugenol improved islet size and reduced necrotic cells, inflammatory cells, and disturbed islets in the pancreas of animals subjected to streptozotocin-induced diabetes. They revealed that eugenol administration reduced cyclooxygenase-2 (COX-2) and increased the expression of peroxisome proliferator-activated receptor alpha (PPAR-α) genes. According to the authors, these results are related to the antioxidant activity of eugenol and its effect on modulating the lipid profile [27].

Type 2 diabetes mellitus can lead to loss of muscle mass due to increased levels of inflammatory mediators [56,57]. Along this line, Jiang et al. (2022) [28] showed that eugenol markedly reduced IL-1, IL-6, and TNF-α concentrations in the skeletal muscle of diabetic mice. The authors also reported that interleukin-10 (IL-10) levels were increased after treatment with eugenol, indicating its modulatory action on the muscle inflammatory response. Corroborating this, animals treated with eugenol had reduced NF-κB expression compared to diabetic mice. These effects of eugenol culminated in the reduction in muscle atrophy, a potential effect of eugenol against the deleterious effects caused by type 2 diabetes mellitus on skeletal muscle [28].

In line with this, Gojani et al. (2023) [29] demonstrated that eugenol decreased pancreatic β-cell loss induced by high glucose-high lipid conditions in vitro. The results revealed that this effect was mediated by the suppression of NLRP3 inflammasome assembly and inhibition of NF-κB activation, resulting in reduced release of IL-1β and TNF-α by macrophages, respectively. These findings suggest that eugenol may act to maintain β-cell structure and function in the context of important metabolic alterations, such as type 2 diabetes.

Recently, Al-Trad et al. (2019) [30] showed that eugenol had a protective effect against high-fat diet and streptozotocin-induced diabetes in rats. The authors attributed this effect to increased insulin sensitivity and glucose uptake in skeletal muscle through the AMP-activated protein kinase/glucose transporter protein type-4 (AMPK/GLUT4) signaling pathway induced by eugenol. It was found that the serum concentration of IL-6 was significantly reduced in diabetic rats treated with eugenol, demonstrating an anti-inflammatory effect. The results of oxidative stress also showed that eugenol had an effective antioxidant action, as it restored serum levels of GSH and MDA, highlighting the association between antioxidant and anti-inflammatory effects for the antidiabetic activity of eugenol [30].

Using islets of Langerhans isolated from rat pancreases, Oroojan et al. (2020) [31] examined the antioxidant activity of eugenol against H_2_O_2_-induced oxidative stress. Analysis of MDA levels revealed that eugenol attenuated islet lipid peroxidation, presenting high serum total antioxidant capacity (T-AOC). Incubation with eugenol also significantly increased CAT, but not SOD, activity in the islets of Langerhans compared to H_2_O_2_ [31]. This finding can be explained by the enzymatic function of CAT in catalyzing the decomposition of H_2_O_2_ into H_2_O and O_2_, thus demonstrating the crucial role of eugenol in neutralizing free radicals.

### 2.6. Eugenol Promotes an Antioxidant Effect and Improves the Inflammatory Response in Infectious Diseases

The immune system is strongly modulated by oxidative stress and pathogen-induced inflammatory processes [58]. In an infection, whether caused by viruses, bacteria, fungi, or protozoa, immune cells adapt their response to secrete specific molecules that generate inflammation and oxidative stress as a way to combat the invading agent [59]. However, adequate control of this process is essential to reduce the risk of secondary complications mediated by an excessive inflammatory response.

According to Paidi et al. (2021) [32], eugenol may be useful in treating inflammation associated with severe acute respiratory syndrome coronavirus 2 (SARS-CoV-2). Experimentally, eugenol reduced NF-κB activation and IL-6, IL-1β, and TNFα expression induced by the Spike S1 protein of SARS-CoV-2 in human lung cells. The authors also revealed that mice infected with SARS-CoV-2 and treated with eugenol had reduced lung inflammation, reduced fever, and improved cardiac function and locomotor activity. In addition, animals treated with eugenol 5 days after infection showed a reduction in the expression of some pro-inflammatory molecules in the lung, culminating in a reduction in symptoms and complications [32].

Yu and colleagues (2022) [33] investigated the therapeutic effect of eugenol on *Aspergillus fumigatus* fungal keratitis in mice. The histological assessment showed that eugenol reduced the infiltration of inflammatory cells and fungal burden 3 days after infection, accompanied by reduced mRNA expression of IL-1β, TNF-α, and iNOS. In another experimental design, eugenol downregulated pro-inflammatory cytokines in the cornea and inhibited IL-1β, IL-6, and IL-8 expression in HCECs stimulated with *A. fumigatus* hyphae. The authors also revealed that eugenol improves fungal keratitis by activating the nuclear factor erythroid 2-related factor 2/heme oxygenase-1 (Nrf2/HO-1) pathway, which plays an important role in cytokine production. The mechanism of antifungal action of eugenol is associated with damage to the membrane of microorganisms and the inhibition of ergosterol biosynthesis, reinforcing its potential application as a therapeutic alternative for the management of fungal keratitis [33].

Zheng and colleagues (2023) [34] investigated the effects of eugenol against infection caused by *Salmonella enterica* in broilers. The researchers showed that eugenol reduced the mRNA levels of myeloid differentiation factor 88 (MyD88), toll-like receptor-4 (TLR4), and iNOS, and suppressed the phosphorylation of p65 and IκBα, as well as the mRNA levels of TNF-α, IL-1β, IL-2, and IL-18 in the cecum. Pro-inflammatory cytokines are linked to the TLR4/MyD88/NF-κB signaling pathway [60], while Gram-negative bacteria release LPS, an endo-inflammatory toxin that is recognized by TLR4. This interaction process activates TLR4, which recruits a MyD88 factor, forming a signaling complex, triggering NF-kB activation [61]. NF-κB consists of P50 (NF-κB1), RelA (P65) or P52 (NF-κB2), and RelB, leading to the release of inflammatory factors. Eugenol also improved histopathological and ultrastructural changes in the cecum, reduced the number of *S. enterica*, contained Proteobacteria and Ruminococcus, and preserved the ratio of Firmicutes to Bacteroidetes. In addition, eugenol sustained the expression of zonula occludens-1 (ZO-1), claudin-1, and tight junction occlusion proteins. Thus, eugenol could protect broilers against *S. enterica* infection by maintaining intestinal microbiota homeostasis and tight junctions involved in mucosal barrier function, as well as limiting massive inflammation [34].

Eugenol also had an antioxidant effect on *Plasmodium berghei*-induced infection in mice. Suleiman et al. (2023) [35] showed that eugenol attenuated the variation in packed cell volume (PCV), a physiological marker for the anemic state of animals, and prevented changes in the histological structure of critical organs in the pathogenesis of malaria, such as the liver, brain, and spleen. The authors also demonstrated that eugenol reduced TBARS levels and increased GSH levels in both organs, demonstrating a crucial role against lipid peroxidation and the consumption of antioxidant defense associated with *P. berghei* infection. In contrast, only eugenol at 10 mg/kg was effective in restoring SOD activity in the liver and brain, but not in the spleen. These findings suggest that eugenol has therapeutic potential to combat anemia and oxidative damage induced by *P. berghei*, revealing a particularly promising role against malaria.

### 2.7. Eugenol Modulates Signaling Pathways During the Immune Response

The involvement of intracellular messengers in response to inflammation or high levels of ROS has been documented in studies using different polyphenols [62]. The blockage or activation of signaling pathways leads to a coordinated modulation of gene transcription, production of effector proteins, or the activity of enzymes important for the control of inflammatory and oxidative reactions [63]. Therefore, the elucidation of the mechanisms linked to the effects of new bioactive molecules is essential for a good understanding of their therapeutic properties.

During inflammation, the complement system recruits neutrophils through the C5a fragment and formyl-Met-Leu-Phe (fMLF). Once stimulated, these cells promote activation of the NADPH oxidase complex, which leads to the formation of two complexes: a membrane-bound complex composed of gp91phox and p22phox proteins, and other cytoplasmic formed by p40phox, p47phox, and p67phox. These protein complexes function as catalysts in the redox reaction, allowing the transfer of electrons from NADPH to oxygen, forming superoxide, a species that precedes the formation of other ROS [36]. 

In this scenario, researchers stimulated ROS production with fMLF in isolated neutrophils incubated with eugenol at different concentrations. The results showed that eugenol inhibited the formation of ROS in a concentration-dependent manner, with an IC50 value of 5 µg/mL. Eugenol also reduced the p47phox phosphorylation, a protein involved in the cytoplasmic complex. When the cytosolic complex does not translocate to the membranes, NADPH oxidase is not activated, thus allowing the reduction in ROS formation. Eugenol also inhibited Raf/MEK/ERK cascade, reducing superoxide formation [36].

In another study, Li and colleagues (2022) [37] demonstrated the antioxidant effects of eugenol in vitro by reducing H_2_O_2_-induced tendon stem cell apoptosis and increasing CAT and SOD expression. The authors revealed that the cytoprotective action of eugenol against oxidative stress is mediated by the modulation of the Nrf2/HO-1 signaling, as pretreatment with ML385, an Nrf2 inhibitor, attenuated the cytoprotective effects of eugenol. This signaling pathway plays a crucial role in preventing cellular and tissue damage caused by oxidative stress, as it controls the transcription of genes related to antioxidant defenses. Therefore, the positive regulation of the Nrf2/HO-1 pathway by eugenol is a promising mechanism for its use as a cytoprotective agent [37]. Interestingly, Ma et al. (2021) [38] found similar results using HEK-293 and NIH-3T3 cells. They observed that eugenol increased the concentration of Nrf2 and its transcriptional activity in both cells, in addition to stabilizing its intracellular levels and preventing its ubiquitination. Subsequently, the antioxidant effect of eugenol was demonstrated using oxidative stress induced by H_2_O_2_, highlighting that Nrf2 is required for the performance of the cytoprotective effect of eugenol [38].

Recently, a study by Jiang et al. (2024) [39] showed that the Nrf2 pathway is also crucial for the antidiabetic activity of eugenol in vivo. Using the streptozotocin-induced type 1 diabetes model, the authors found that eugenol increased the expression of Nrf2-regulated HO-1 and NAD(P)H quinone dehydrogenase 1 (NQO-1), and that this effect led to reduced β-cell damage. In addition, eugenol also increased insulin secretion and reduced the expression of apoptotic and oxidative stress markers, which may contribute to the protection of pancreatic β-cells. In contrast, a reversal of this effect was observed after use of the Nrf2 inhibitor ML385, indicating the relevance of the Nrf2-mediated pathway for the protection provided by eugenol [39].

## 3. Conclusions

The data show that eugenol exerts beneficial effects on ameliorating oxidative stress and its main actions are related to anti-inflammatory properties. Although these processes are not completely understood, the mechanisms of action of eugenol involve the modulatory effects of different signaling pathways. The dual anti-inflammatory and antioxidant actions, commercial availability, and abundance of this compound make it an interesting target for advances in toxicological and clinical studies aimed at demonstrating its potential as a promising therapeutic agent in a variety of conditions in which inflammation and oxidative stress play a dominant pathological role.

## Figures and Tables

**Table 1:** Eugenol modulates the anti-inflammatory and antioxidant response in joint, lung, and liver.

Experimental Protocol	Animals and/or Cell Lines	Eugenol	Biological Effect	Reference
TNF-α-induced fibroblast-like synoviocytes	Human fibroblast-like synovial cell (HFLS)	5, 10 or 20 μM	Inhibited TNF-α-induced proliferation, migration, invasion, angiogenesis, inflammation and promoted apoptosis	[11]
Rheumatoid arthritis	Human peripheral blood mononuclear cells	10, 20 or 40 µM	Reduced NO, TNF-α and IL-6 levels, as well as improved oxidative stress markers	[12]
Collagen-induced arthritis	Wistar rats	10 or 20 mg/kg	Decreased ROS, NO, oxidation markers and increased enzymatic and non-enzymatic antioxidants. The levels of TNF-α, IL-6, and IL-10 were also ameliorated	[13]
Collagen-induced arthritis	Wistar rats	Nanoparticles packed with eugenol	Decreased MDA, TGF-β, MCP-1 and FOXO3 expression	[14]
Carrageenan-induced arthritis	Wistar rats	2.5 to 10 mg/kg	Increased SOD, CAT, GSH, GPx, and GSH S-transferase activities	[15]
Cigarette smoke-induced lung injury	C57BL/6 mice	100 mg/mL	Reduced lung morphological changes, inflammation, and oxidative stress markers, while increasing IL-10	[16]
Lipopolysaccharide-induced lung inflammation	RAW 264.7 macrophages and BALB/c mice	10–60 μg/mL and 10–60 mg/kg	Reduced NO, IL-1β, IL-6, lung edema, collagen content, and inflammatory cell infiltration (including positive for iNOS, MMP-9, and TIMP-1). Inhibition of JNK phosphorylation	[17]
Lipopolysaccharide-induced inflammation	IPEC-J2 cells	100 μM	Suppressed IL-8 and TNF-α levels, while restored ZO-1, occludin, and nutrient transporters	[18]
Lipopolysaccharide-induced lung injury	BALB/c mice	15 mg/kg to 1500 mg/kg	Inhibited inflammatory cytokines release (TNF-α, IL-1β, and IL-6), NADPH oxidase, and antioxidant enzymes activity (SOD, CAT, and GPx)	[19]
Carbon tetrachloride-induced liver injury	Wistar rats	10 mg/kg or 100 mg/kg	Improved hepatic histopathological changes, reduced serum aminotransferases, inflammation and oxidative stress	[20]
Hepatic steatosis and hypercholesterolemia	Wistar rats	10 or 100 mg/kg	Reduced total cholesterol, LDL, atherogenic index, steatosis, hepatic inflammation, ALT, ALP, increased SOD and CAT activity, and downregulated TRPV1	[21]
CdCl_2_-induced hepatic inflammation	Wistar rats	3 or 5 mg/kg	Reversed LDH, GGT, ALP, ALT, AST, and bilirubin level. Reduced oxidative stress and inflammatory markers	[22]

Abbreviations: ALP, alkaline phosphatase; ALT, alanine transaminase; AST, aspartate aminotransferase; CAT, catalase; GGT, gamma glutamyl transferase; GPx, glutathione peroxidase; GSH, glutathione; IL-1, interleukin; iNOS, inducible nitric oxide synthase; LDH, lactate dehydro-genase; LDL, low density lipoproteins; MCP-1, monocyte chemoattractant protein-1; MDA, malondialdehyde; MMP-9, matrix metalloproteinase-9; NO, nitric oxide; ROS, reactive oxygen species; SOD, superoxide dismutase; TGF-β, transforming growth factor-beta; TIMP-1, tissue inhibitor of metalloproteinases-1; TNF-α, tumor necrosis factor-alpha; TRPV1, transient receptor potential vanilloid 1; ZO-1, zonula occludens-1.

**Table 2:** Eugenol improves the inflammatory response and oxidative stress in neuronal damage, diabetes, and infection models.

Experimental Protocol	Animals and/or Cell Lines	Eugenol	Biological Effect	Reference
Streptozotocin-induced diabetic neuropathy	Sprague Dawley rats	10 mg/kg	Decreased NGF concentrations, oxidative stress, and TNF-α levels	[23]
Pilocarpine-induced status epilepticus	Wistar rats	200 mg/kg	Reduced apoptotic neuronal cell death, activation of astrocytes and microglia, expression of IL-1β and TNF-α, inhibited NF-κB activation and the formation of the NLRP3 inflammasome in the hippocampus	[24]
6-OHDA-induced oxidative stress	Wistar rats	0.1, 1.0 or 10 mg/kg	Reduced MDA and NO levels in the brain, restored GSH levels, improved gene expression related to antioxidant defenses (SOD, CAT, and GPx) and neurotrophic factors (BDNF, Ntrk2, GFRα1, and GDNF)	[25]
AChE, BChE, and MAO in vitro activities	Albino rats	100 µM	Scavenged ABTS radicals and a high ferric reducing power, suppressed the increased TBARS levels, and inhibited AChE and MAO activities	[26]
Streptozotocin-induced diabetes	Wistar rats	12 or 24 mg/kg	Reduced serum glucose, total cholesterol, triglyceride, LDL, AST, ALT, MDA, pancreas necrosis, and COX-2 expression. Increased insulin levels, HDL, SOD, and PPAR-α expression	[27]
High-fat diet/streptozotocin-induced diabetes	C57BL/6N mice	10 or 20 mg/kg	Increased GLUT4 translocation and AMPK phosphorylation in skeletal muscle, intracellular Ca^2+^ via silencing TRPV1 gene, activated CaMKK2, and reduced AMPK phosphorylation	[28]
Lipopolysaccharide-induced inflammation and high glucose-high lipid conditions-induced cell damage	THP-1 macrophages and INS-1 832/13 rat insulinoma cells	5, 10 or 15 µM	Reduced production of TNF-α and IL-1β via inhibition of NF-κB activation and suppression of NLRP3 inflammasome assembly. Decreased dedifferentiation and β -cell loss	[29]
High-fat diet/streptozotocin-induced diabetes	Sprague Dawley rats	10 mg/kg	Reduced serum glucose, triglyceride, total cholesterol, LDL, MDA, IL-6, insulin, and glutathione, while increasing contents of GLUT4 and AMPK in skeletal muscle	[30]
H_2_O_2_-induced oxidative stress	NMRI mice	50, 100 or 200 μM	Reduced lipid peroxidation and increased T-AOC. Eugenol also increased CAT, but not SOD, in islets of Langerhans	[31]
SARS-CoV-2 spike S1 with ACE2 binding and SARS-CoV-2 Spike S1-induced COVID-19 phenotype	C57BL/6 mice	25 mg/kg	Inhibited the interaction between spike S1 and ACE2. Reduced SARS-CoV-2 spike S1-induced activation of NF-κB and inflammatory markers expression. Reduced lung inflammation, fever, and improved heart function	[32]
*Aspergillus fumigatus*-induced fungal keratitis	HCEC cells and C57BL/6 mice	80 or 160 µg/mL	Reduced IL-1β, TNF-α and iNOS mRNA expressions. In vitro, inhibited IL-1β, IL-6 and IL-8 expressions, activating the Nrf2/HO-1 signaling pathway	[33]
*Salmonella enterica* infection	Broiler chickens	25 to 50 mg/kg	Reduced of MyD88, TLR4 and iNOS mRNA levels. It also suppressed p65 and IκBα phosphorylation, as well as TNF-α, IL-1β, IL-2 and IL-18 mRNA levels in cecum	[34]
*Plasmodium berghei*-induced anaemia and oxidative organ damage	Swiss mice	10 or 20 mg/kg	Reduced TBARS levels and increased GSH levels, and restored SOD activity in the liver and brain	[35]
fMLF-induced superoxide anion production	Human neutrophils	2.5 µg/mL to 20 µg/mL	Inhibited superoxide anion generation by neutrophils via inhibition of Raf/MEK/ERK1/2/p47phox pathway	[36]
Rat patellar tendon injury and in vitro antioxidant effects	Sprague Dawley and Tendon stem cells	50 µM	Increased CAT and SOD expression, and promoted cytoprotective effect mediated by Nrf2/HO-1 pathway	[37]
H_2_O_2_-induced cell damage	HEK-293 and NIH-3T3 cells	0 to 100 µg/mL	Increased Nrf2 concentration and its transcriptional activity, in addition to stabilizing its intracellular levels and preventing its ubiquitination	[38]
Streptozotocin-induced diabetes	C57BL/6N mice and MIN6 pancreatic β-cell line	5, 10 or 15 mg/kg	Increased activation of Nrf2 and Nrf2-regulated expression of NQO-1 and HO-1. Reduced cell damage. Increased insulin secretion and reduced expression of apoptotic and oxidative stress markers	[39]

Abbreviations: ACE2, angiotensin converting enzyme 2; AChE, acetylcholinesterase; ALT, alanine transaminase; AST, aspartate aminotransferase; AMPK, AMP-activated protein kinase; BDNF, brain-derived neurotrophic factor; CaMKK2, calcium/calmodulin-dependent protein kinase 2; CAT, catalase; COVID-19, coronavirus disease 2019; COX-2, cyclooxygenase-2; GDNF, glial cell derived neurotrophic factor; GFRα1, GDNF family receptor alpha-1; GLUT4, glucose transporter protein type-4; GSH, glutathione; GPx, glutathione peroxidase; HDL, high density lipoprotein; H_2_O_2_, hydrogen peroxide; HO-1, heme oxygenase-1; IL, interleukin; iNOS, inducible nitric oxide synthase; IκBα, nuclear factor of kappa light polypeptide gene enhancer in B-cells inhibitor-alpha; MAO, monoamine oxidase; MDA, malondialdehyde; MyD88, myeloid differentiation factor 88; NF-κB, nuclear factor-kappa B; Nfr2, nuclear factor erythroid 2-related factor 2; NGF, nerve growth factor; NO, nitric oxide; NQO-1, NAD(P)H quinone dehydrogenase 1; Ntrk2, neurotrophic receptor tyrosine kinase 2; PPAR-α, peroxisome proliferator-activated receptor alpha; SARS-CoV-2, severe acute respiratory syndrome coronavirus 2; SOD, superoxide dismutase; T-AOC, total antioxidant capacity; TBARS, thiobarbituric acid reactive substances; TLR4, Toll-like receptor-4; TNF-α, tumor necrosis factor-alpha; TRPV1, Transient receptor potential vanilloid 1.
